# PATIENT PREFERENCES FOR LEVEL OF HEALTH LITERACY IN APPS FOR CHRONIC DISEASE SELF-MANAGEMENT

**DOI:** 10.1093/geroni/igz038.3488

**Published:** 2019-11-08

**Authors:** Mary Louise Goodyear, Juan J Toral-Garcia, Amarilis Acevedo, Drenna Waldrup-Valverde, Raymond Ownby

**Affiliations:** 1 Department of Psychiatry and Behavioral Medicine, Nova Southeastern University, Fort Lauderdale, Florida, United States; 2 Nova Southeastern University, Fort Lauderdale, Florida, United States; 3 Nell Hodgson Woodruff School of Nursing, Atlanta, Georgia, United States

## Abstract

In spite of expert recommendations that written material should be provided at a level of health literacy that matches that of the person receiving it, there have been few studies of matching. In this study we evaluated the utility of a new strategy to assess patients’ preference for information at different difficulties and assessed the relation of their preference to measured health literacy and health locus of control (LOC). We measured health literacy in participants then asked them to choose between pairs of texts with the same content but at the 3rd, 6th, or 8th-grade levels. Statistical analyses assessed the relation of participants’ health literacy to their preference as well as personal characteristics. Participants (n = 155) were 84 women and 71 men aged from 40 to 82 years (mean=57; 136 African Americans and 19 whites). Participants had clear preferences: those with lower levels of health literacy preferred texts at the 3rd grade level and those with higher levels preferred more difficult texts. Preference was not related to age, gender or race but was to education and health literacy (p < 0.05). Persons who chose more difficult texts reported higher levels of internal health locus of control (t [144] = 2.68, p = 0.01). A predictive analytic model using education and preference resulted in 80% correct classification of participants. Using this model may be a simple way to match information presentation to patients’ level of health literacy. Further research on this strategy is needed.

